# Multi‐Color, Bleaching‐Resistant Super‐Resolution Optical Fluctuation Imaging with Oligonucleotide‐Based Exchangeable Fluorophores

**DOI:** 10.1002/anie.202013166

**Published:** 2021-02-03

**Authors:** Marius Glogger, Christoph Spahn, Jörg Enderlein, Mike Heilemann

**Affiliations:** ^1^ Institute of Physical and Theoretical Chemistry Goethe-University Frankfurt Max-von-Laue Str. 7 60438 Frankfurt Germany; ^2^ Third Institute of Physics—Biophysics Georg August University 37077 Göttingen Germany; ^3^ Cluster of Excellence “Multiscale Bioimaging: from Molecular Machines to Networks of Excitable Cells” (MBExC) Georg August University Göttingen Germany

**Keywords:** DNA-PAINT, fluorescence, photobleaching, SOFI, super-resolution microscopy

## Abstract

Super‐resolution optical fluctuation imaging (SOFI) is a super‐resolution microscopy technique that overcomes the diffraction limit by analyzing intensity fluctuations of statistically independent emitters in a time series of images. The final images are background‐free and show confocality and enhanced spatial resolution (super‐resolution). Fluorophore photobleaching, however, is a key limitation for recording long time series of images that will allow for the calculation of higher order SOFI results with correspondingly increased resolution. Here, we demonstrate that photobleaching can be circumvented by using fluorophore labels that reversibly and transiently bind to a target, and which are being replenished from a buffer which serves as a reservoir. Using fluorophore‐labeled short DNA oligonucleotides, we labeled cellular structures with target‐specific antibodies that contain complementary DNA sequences and record the fluctuation events caused by transient emitter binding. We show that this concept bypasses extensive photobleaching and facilitates two‐color imaging of cellular structures with SOFI.

## Graphical abstract

Fluorophore labels that transiently bind to and unbind from a protein target allow photobleaching in super‐resolution optical fluctuation imaging (SOFI) to be circumvented. Using short fluorophore‐labeled DNA oligonucleotides, we demonstrate high‐contrast, low‐background, and multi‐color super‐resolution imaging with SOFI.

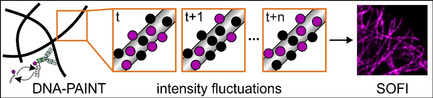

Super‐resolution microscopy has significantly improved our understanding of cell biology.^[1]^ Among these are methods that build on the detection of stochastic single‐molecule emission events, which are subsumed under the term single‐molecule localization microscopy (SMLM) and that allow for imaging of cellular structures with nanometer resolution.^[2]^ High resolution is achieved by spatiotemporal separation of emission events via fluorophore “blinking” and precise localization of individual molecules. Alternatively, transient and reversible binding of fluorophore labels enables the isolation of single emitters in space and time, as for example, in points accumulation for imaging in nanoscale topography (PAINT)^[3]^ and DNA‐PAINT.^[4]^

An alternative and elegant way to generate super‐resolved images is provided by super‐resolution optical fluctuation imaging (SOFI). SOFI is a software‐based method of calculating resolution‐enhanced images from a time series of diffraction‐limited images and provides a useful trade‐off between temporal and spatial resolution.^[5]^ In SOFI, contrast‐enhanced and background‐reduced high‐resolution images are obtained by a correlation analysis of intensity‐time traces. The method requires only the labeling of a sample with stochastically fluctuating fluorescent emitters, is compatible with a variety of imaging platforms and flexible with respect to imaging conditions.^[6]^ The power of this method has successfully been demonstrated in cells using various fluorophore labels that exhibit photoswitching and lead to a fluctuating fluorescence signal, including quantum dots,^[5]^ organic dyes,^[7]^ or fluorescent proteins.^[8]^ However, the usability of organic dyes and fluorescent proteins for SOFI is limited by their inherent susceptibility to photobleaching, although correction methods for photobleaching in SOFI have been proposed^[9]^ and self‐blinking dyes were shown to increase signal stability.^[10]^ Quantum dots do not have this problem but they are large in size and difficult to use for target‐specific labeling. Hence, it would be desirable to have small fluorophore labels that show adjustable fluorescence fluctuations and, at the same time, enable long acquisition sequences without loss of signal, which would enable higher‐order statistical analysis for SOFI (leading to better resolved images).

Here, we present fluorophore labels that transiently and repetitively bind to a target as probes for SOFI. Transient labels typically show a weak affinity to a target, and exchange constantly with the buffer that constitutes a reservoir (we refer to these labels as “exchangeable labels” hereafter). As such, these labels are insensitive to common photobleaching and thus yield a constant fluorescence signal over time, which has been successfully exploited in SMLM[^3^, ^4^, ^11^] and stimulated emission depletion (STED) microscopy.^[12]^

In order to implement exchangeable labels for SOFI, we built on the concept of DNA‐PAINT, in which stochastic “blinking” is achieved by transient and reversible binding of exchangeable fluorophore‐labeled oligonucleotides (imager strands) from a buffer reservoir to complementary DNA‐sequences (docking strands). Using secondary antibodies for covalent attachment of docking strands (Figure 1 A) allows highly specific and multiplexed imaging of cellular structures with high spatial resolution.^[13]^ Because of a permanent replenishment of imager strands, DNA‐PAINT is little susceptible to photobleaching and hence allows for theoretically infinitely long acquisition times.

**Figure 1:**
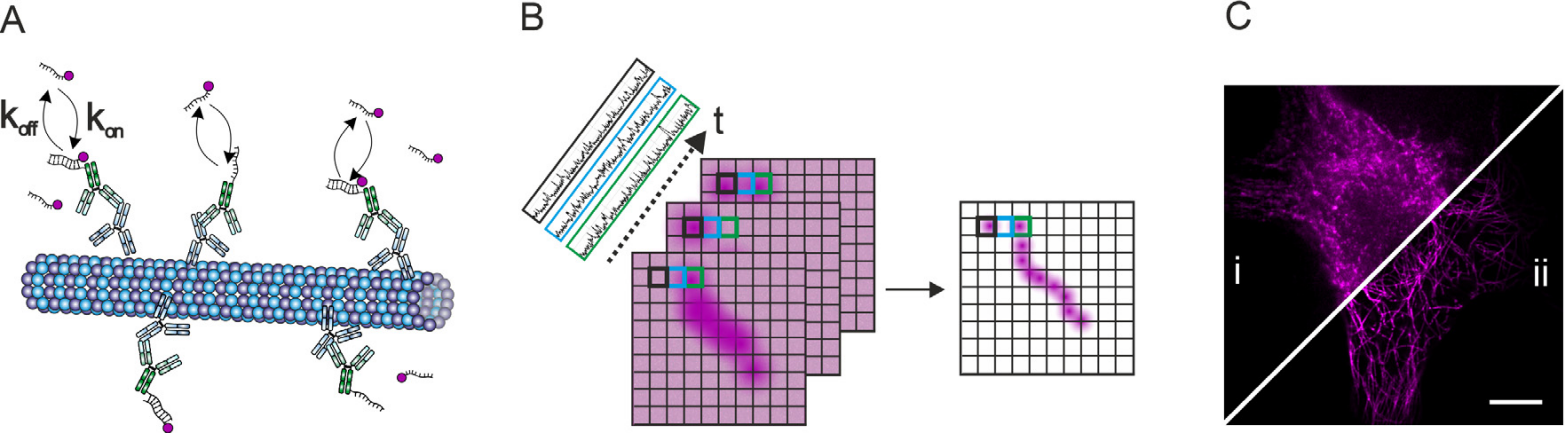
Principle of SOFI with oligonucleotide‐based exchangeable fluorophore labels. A) Labeling of cellular structures using oligonucleotide‐conjugated (docking strands) secondary antibodies and sequence‐complementary, fluorophore‐labeled oligonucleotides (imager strands). Transient and reversible binding of imager strands causes target‐specific intensity fluctuations. B) Image processing in SOFI via time‐trace analysis of stochastically fluctuating emitters. SOFI images are resolution and contrast enhanced and exhibit reduced unspecific background signals. C) Diffraction limited (i) and resolution‐enhanced 2^nd^ order SOFI image (ii) of a microtubule‐labeled U2‐OS cell using P1‐AbberiorStar635P as exchangeable imager strand (scale bar 10 μm).

We exploit the transient and reversible binding of imager strands to DNA‐labeled antibodies to generate target‐specific and bleaching‐resistant “blinking” events required for SOFI analysis (Figure 1 B). We first tested the suitability of exchangeable, dye‐labeled oligonucleotides for SOFI by immunofluorescence labeling of microtubules with primary antibodies against the β‐tubulin subunit and suitable docking‐strand labeled secondary antibodies. In a titration experiment we applied different imager strand concentrations (P1‐AbberiorStar635P) to generate the site‐specific intensity fluctuations required for the SOFI correlation analysis and found imager strand concentrations in the lower nanomolar range to be most suitable (Figures 1 C, S1). While in unprocessed images, target‐specific and background signals are difficult to discriminate due to a large amount of freely diffusing imager strands, the background signal was efficiently removed in 2^nd^ order SOFI images, and fine microtubule structures became visible (Figure 1 C). This is because in SOFI, uncorrelated background signal is efficiently eliminated. Using higher or lower imager strand concentrations reduced the quality of the processed images, probably due to low numbers of “blinking” events or quasi‐permanent signals, respectively (data not shown). We found the imager strand concentrations in the range of 5 to 20 nm well suited for SOFI analysis, as they ensure an optimal level of intensity fluctuations and at a same time a good signal‐to‐background ratio. We recommend using this concentration range as a starting point, and to eventually optimize this parameter in new experiments, for example, for different DNA sequences or target structures.

We next estimated the resolution enhancement in SOFI images by applying an image decorrelation analysis as described by Descloux et al.^[14]^ We therefore imaged and analyzed U2‐OS cells immunolabeled for β‐tubulin. We compared image resolution after decorrelation analysis of mean intensity time series projections (395±18 nm), 2^nd^ order (285±14 nm), and 3^rd^ order (218±14 nm) SOFI images. The resolution enhancement is in good agreement with theoretical estimates, predicting a spatial resolution that is proportional to the square‐root of the SOFI correlation order.^[5]^ Since the spatial resolution reported by the decorrelation analysis might be impacted by background signal from freely diffusing imager strands, we performed a second analysis by quantifying the intensity profile across the microtubule axis as a metric for image resolution. Determination of the mean microtubule diameters (FWHM) revealed 329±50 nm, 245±25 nm and 205±29 nm for the mean intensity time series projection, 2^nd^ order and 3^rd^ order SOFI image, respectively (Figure 2 A). As a third approach and to access the local resolution in our images, we performed Fourier‐ring‐correlation (FRC) analysis using the NanoJ Fiji package.^[15]^ The resulting FRC maps show a homogenous distribution of FRC values in cell regions, and resolution enhancement correlates well with decorrelation analysis and tubulin measurements (Figure S2).

**Figure 2:**
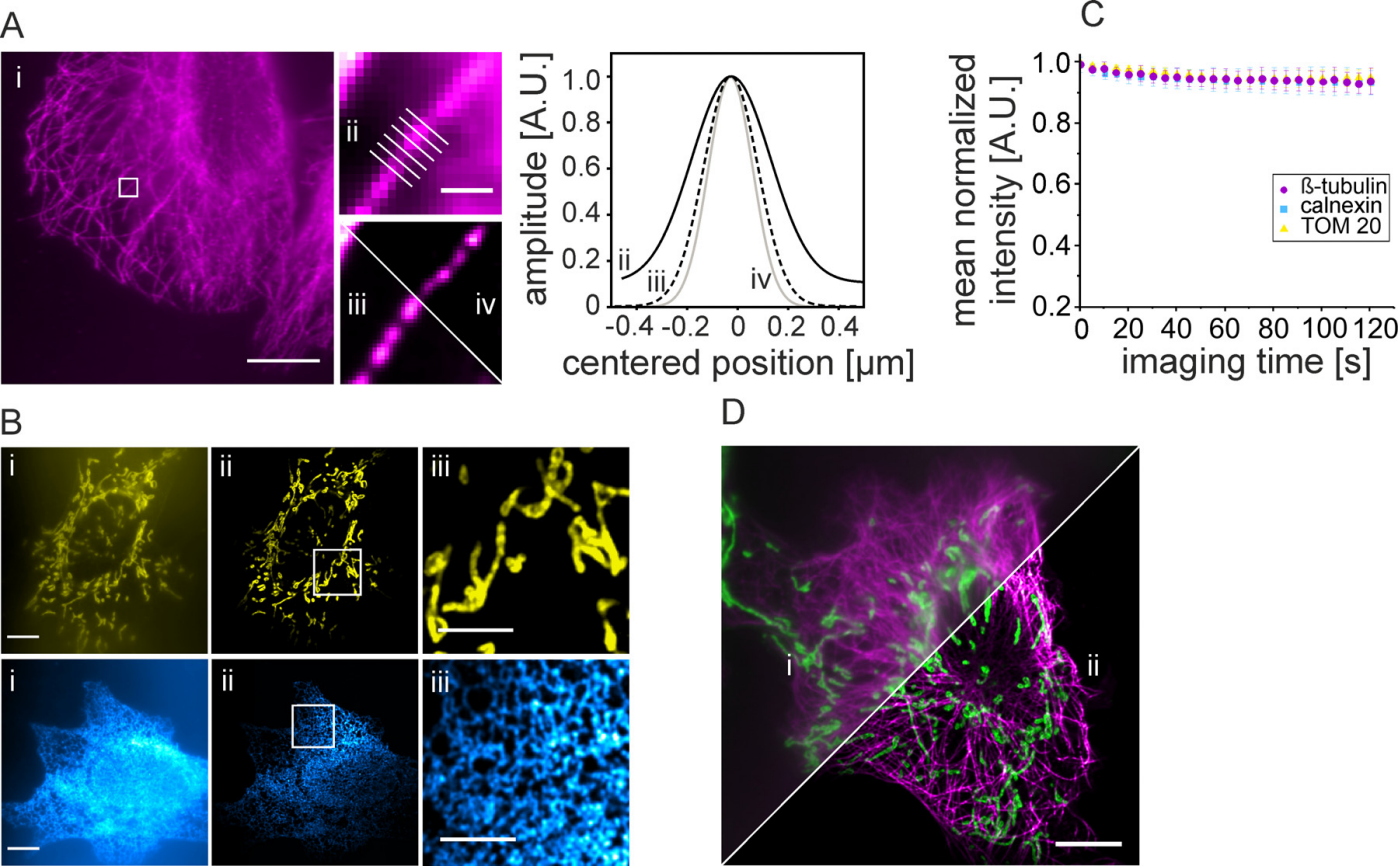
SOFI imaging of various cellular structures using DNA‐based exchangeable fluorophore labels. A) Quantification of resolution enhancement in SOFI images. i)  Diffraction‐limited mean‐intensity time series projection of a microtubule‐labeled U2‐OS cell. ii) Magnified region shown in (i) and corresponding to 2^nd^ (iii) and 3^rd^ order (iv) SOFI images (scale bars are 10 μm (i) and 1 μm (ii–iv)). Determination of full‐width‐at‐half‐maxima (FWHM) of intensity profiles across microtubules as a metric for image resolution in magnified regions of diffraction‐limited (ii, black solid line), 2^nd^ order SOFI (iii, black dashed line), and 3^rd^ order SOFI (iv, grey solid line) images. The FWHM values are 329±50 nm (ii), 245±25 nm (iii), and 205±29 nm (iv). B) Diffraction‐limited mean‐intensity time series projection (i) and 2^nd^ order SOFI images (ii) of mitochondria (upper row, TOM20, yellow) and the endoplasmic reticulum (lower row, calnexin, blue) with corresponding magnified regions shown in (iii) (scale bars are 10 μm (i and ii) and 5 μm (iii)). C) Fluorescence signal versus time recorded on a widefield microscope during SOFI experiments with exchangeable oligonucleotides (P1‐AbberiorStar635P). Labeled are microtubules (β‐tubulin, magenta, 20 nm), the endoplasmic reticulum (calnexin, blue, 20 nm), and mitochondria (TOM20, yellow, 5 nm). Shown are mean intensities ± standard deviation of intensity signals from >20 cells. D) Two‐color SOFI with exchangeable oligonucleotides. Shown is a diffraction‐limited mean‐intensity time series projection (i) and 2^nd^ order SOFI image (ii) of microtubules (β‐tubulin, magenta) and mitochondria (TOM20, green) (scale bar 10 μm).

To demonstrate the versatility of exchangeable labels for SOFI, we labeled two other cellular structures and generated SOFI images. Mitochondria and the endoplasmic reticulum were labeled by targeting the outer membrane protein TOM20 and calnexin, respectively (Figure 2 B). In 2^nd^ order SOFI images, the background signal is strongly reduced, and the resolution enhancement allowed the visualization of the structural features associated with the intracellular membrane structures. On this imaging data, we quantified the degree of photobleaching occurring during SOFI experiments with exchangeable fluorophores. Under the experimental settings applied (see materials and methods), we find a rather constant fluorescence signal over time for the whole imaging time series (Figure 2 C). For comparison with a permanent label, we recorded a fluorescence time series of Alexa Fluor 647 labeled β‐tubulin using dSTORM photoswitching conditions, resulting in a significant loss of signal over time (Figure S3). We hypothesize that continuous and fast exchange of imager strands via recurring exchange cycles prevents target specific photobleaching. This allows long image acquisition times and bypasses the need for correcting a decrease in signal intensities in SOFI.^[9]^ It would further be interesting to explore higher‐order SOFI reconstructions, and thus obtain a higher spatial resolution. This will require an analysis that avoids over‐representation of high‐intensity regions which yield a stronger correlation signal than low‐intensity regions. In our hands, we found 2^nd^ and 3^rd^ order a very good compromise between contrast and resolution enhancement, signal conservation, straight‐forward SOFI analysis and computational time for image reconstruction.

We finally extended the concept of SOFI with exchangeable oligonucleotides to two‐color imaging. We labeled microtubules and mitochondria in the same cell and performed consecutive imaging in different spectral channels using suitable target‐specific imager strands (Figure 2 D, P1‐AbberiorSTAR 635P and P4‐Cy3b, respectively). In agreement with the results presented previously, both imaging channels exhibited less background signal and showed an increased resolution after image processing. SOFI only depends on stochastic “blinking” and is independent of spectral properties. Thus, the concept can easily be extended to multi‐color imaging by for example, using multiple spectrally distinct imager strands.^[13]^

In this work, we introduce oligonucleotide‐based exchangeable fluorophore labels for bleaching‐insensitive SOFI. Recurring, target‐specific and fast exchange cycles of dye‐labeled DNA generate side‐specific intensity fluctuations that can be used for higher order SOFI statistical analysis. Image processing generates background‐reduced and resolution‐enhanced images. Compared to DNA‐assisted SMLM that typically uses a concentration of imager strands in the range of 1 nm, SOFI demands for a higher imager strand concentration (5–20 nm in this work) to generate sufficient site‐specific fluctuation events (Figure 3). This leads to a higher background in fluorescence images originating from freely diffusing imager strands, which however is successfully removed by the SOFI analysis. The inherent flexibility of DNA‐PAINT with respect to oligonucleotide design allows straight‐forward manipulation of binding kinetics^[16]^ and thus to adjust the imager strand concentration as well as the imaging speed. Furthermore, the usage of other protein‐specific DNA labels such as DNA‐based aptamers^[17]^ are an attractive labeling alternative for SOFI imaging of cellular structures. Imaging multiple targets with SOFI profits from the sequence flexibility of DNA‐assisted exchangeable probes.^[13]^ In summary, SOFI with exchangeable fluorophores provides a useful trade‐off between temporal and spatial resolution, can easily be implemented into common microscopy setups and thus expands the repertoire of super‐resolution experiments using exchangeable dyes.

**Figure 3:**
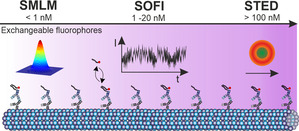
Comparison of super‐resolution imaging methods using exchangeable, dye‐labeled oligonucleotides. The imager strand concentration depends on the imaging method and is typically <1 nm for SMLM, 1–20 nm for SOFI, and >100 nm for STED.

## Conflict of interest

The authors declare no conflict of interest.

## Supporting information

As a service to our authors and readers, this journal provides supporting information supplied by the authors. Such materials are peer reviewed and may be re‐organized for online delivery, but are not copy‐edited or typeset. Technical support issues arising from supporting information (other than missing files) should be addressed to the authors.

Supplementary
