# Supplementary material for: Multi‐Color, Bleaching‐Resistant Super‐Resolution Optical Fluctuation Imaging with Oligonucleotide‐Based Exchangeable Fluorophores
Source: Angew Chem Int Ed Engl. 2021 Feb 3;60(12):6310–3. doi: 10.1002/anie.202013166 (PMC7986781; doi:10.1002/anie.202013166)
Supplement: Supplementary file 1 — Supplementary [file ANIE-60-6310-s001.pdf]

Angewandte Chemie
Eine Zeitschrift der Gesellschaft Deutscher Chemiker

Supporting Information

Multi-Color, Bleaching-Resistant Super-Resolution Optical Fluctuation Imaging with Oligonucleotide-Based Exchangeable Fluorophores

Marius Glogger, Christoph Spahn, Jörg Enderlein, and Mike Heilemann**

anie_202013166_sm_miscellaneous_information.pdf

Supplementary Information

Table of Contents

Experimental Methods 2

Supplementary Tables 7

Supplementary Figures 8

References 11

Experimental Methods

Cell Culture

Experiments were performed in human U-2 OS bone cancer cells (CLS Cell Lines Service GmbH, Germany). Cells were grown in DMEM medium supplemented with 4.5 g/l glucose, 10% v/v fetal bovine serum and 1% v/v GlutaMAX (all purchased from Gibco, Life Technologies, USA) at 37 °C and 5% CO_2. 24 hours before preparation for microscopy, cells were seeded on fibronectin-coated (Sigma Aldrich, Germany, 15 µg/ml) 8-well chamber slides (Sarstedt, Germany) at a density of 0.7-1.5 x 10^4 cells/well.

Sample preparation

Primary antibodies against tubulin (mouse anti-β-tubulin, #32-2600, Thermo Fisher, USA), the endoplasmic reticulum (rabbit anti Calnexin, #abb22595, Abcam, UK) and mitochondria (rabbit anti-TOM20, #sc-11415, Santa Cruz, USA) were used for labeling. For primary antibody labeling custom DNA docking strand-labeled secondary antibodies (AffiniPure rabbit-anti-mouse # 315-005-003 (P1), AffiniPure goat-anti-rabbit # 111-005-00 (P1), AffiniPure donkey-anti-rabbit # 711-005-152 (P4) and AffiniPure goat-anti-mouse # #115-005-003 (P1), all Jackson Immuno Research, USA) were used. DNA-labeled secondary antibodies were prepared as described previously [1]. For *d*STORM experiments Alexa Fluor 647-labeled donkey-anti-rabbit was used as a secondary antibody (# A-31573, Thermo Fisher).

Chemical fixation and immunolabeling

U-2 OS cells were chemically fixed with prewarmed (37°C) 4% methanol-free formaldehyde (FA, Sigma-Aldrich, Germany) and 0.1% glutaraldehyde (Electron Microscopy Sciences, USA) in PHEM buffer [2] for 60 min at room temperature (RT), followed by three washing steps with PBS. Cells were quenched with 0.2% $NaBH_4$ in PBS for 7 min and washed thrice with PBS. Fixed cells were permeabilized and blocked with permeabilization/blocking buffer (PB, 3% IgG-free BSA, 0.1 % saponin, PBS) for 60 min at RT. Subsequently, primary antibodies diluted in PB-buffer were added to the chambers (Supplementary Table 2) and incubated over night at 4°C. Excess primary antibody was removed by multiple washing steps with PB-buffer and PBS followed by incubation with docking strand- or Alexa Fluor 647-labeled secondary

antibodies for 90 min. After removing excess secondary antibodies, cells were post-fixed with 4% FA for 10 min at RT, washed thrice with PBS and quenched with 50 mM NH_4Cl in PBS for 20 min. Finally, cells were washed thrice with PBS and stored in 0.01% NaN_3 (w/v) (Roth, Germany) in PBS at 4°C until used.

Prior to exchange DNA-PAINT SOFI measurements, P1-AbberiorSTAR635P- or P4-Cy3B-labeled imager strands were diluted in imaging buffer (500 mM NaCl in PBS, pH 8.3) and added to the chambers. For *d*STORM-based SOFI experiments, cells were imaged in reducing agent containing buffer (0.1 M MEA in PBS, pH 7.4), supplemented with an oxygen scavenger system consisting of 10 nM protocatechuate-3,4-dioxygenase (PCD, #03930590, Sigma Aldrich, Germany) and 2.5 mM protocatechuic acid (PCA, #P8279, Sigma Aldrich, Germany).

Microscopy setup for SOFI measurements

A home-built microscope setup was used that was previously described in detail [3]. Two diode lasers (532 nm 150 mW or 637 nm 140 mW, Obis, Coherent Inc., Santa Clara, USA) coupled into a single-mode optical fiber (P1-460B-FC-2, Thorlabs, Dachau, Germany) via a fiber collimator (60FC-0-RGBV11-47, Schäfter+Kirchhoff GmbH, Hamburg, Germany) were used for sample illumination. The excitation light was selected via an acousto-optical tunable filter (AOTF, AOTFnC-400.650-TN, AA Opto-Electronic, Orsay, France). For uniform circular field illumination [4], a 2-axis galvo scanner (GVS012/M, Thorlabs, Dachau, Germany) was placed after the fiber output. A telescope system consisting of two lenses with focal lengths of 50 and 100 mm (AC255-050-A-ML and AC508-100-A-ML, Thorlabs) was used for beam expansion and the excitation light was focused on the back focal plane of the lens (UPlanSApo, 100x, NA 1.40, Olympus Deutschland GmbH, Hamburg, Germany). Excitation and emission light were separated via a dichroic mirror and a long pass filter (Dual Line zt532/642rpc, AHF Analysentechnik AG, 532 LP Edge Basic, AHF Analysentechnik AG, Tübingen, Germany). A nosepiece stage (IX2-NPS, Olympus) was used for z-plane adjustment and to minimize z-drift. Fluorescence light was detected using an EMCCD camera (iXonEM+ DU-897, Andor Technology Ltd., UK).
Laser powers of 7 mW and 13 mW (measured at the fiber exit) were used for excitation of AbberiorSTAR 635P and Cy3b conjugated imager strands, respectively. µManager [5] version 1.4.22 was used for camera control and 5000 consecutive frames were

acquired at 40 Hz with an EM-gain of 50, a preamplifier gain of 5.3, and a readout rate of 3 MHz.

Microscopy setup for comparison of permanent and exchangeable label

Image acquisition was performed at the N-STORM super-resolution microscopy system (Nikon, Japan) equipped with an oil immersion objective (Apo TIRF, 100x, NA 1.49, Nikon, Japan) and an EMCCD camera (DU-897U-CS0-#BV, Andor Technology, UK). AbberiorStar635P-conjugated imager strands or Alexa Fluor 647-labeled secondary antibodies were excited with a 647 nm laser beam at an intensity of 1.1 kW/cm^2 (measured at the objective). All measurements were performed in HILO mode. A 405 nm diode laser was used to control emitter density in *d*STORM experiments. For each condition, 5.000 frames were acquired at 40 Hz in active frame transfer mode with an EMCCD gain of 50-200 at an effective pixel size of 158 nm. Software tools NIS Elements (Nikon, Japan), LCControl (Agilent, USA), and Micro-Manager 1.4.22 were used for setup control and data acquisition.

Image analysis

Super-resolution optical fluorescence imaging

Raw images from the acquired image stack were up-sampled (using lossless interpolation by zero-padding of the frequency-limited Fourier-transformed images) to half the physical pixel size to accommodate the final image pixel size to the SOFI-enhanced spatial resolution [6]. Then, for each pixel, we calculated 2nd and 3rd order correlation values C_2 and C_3 over the whole stack of K recorded frames by using the formulas

$$C_2 = \sum_{s=0}^{\lfloor \log_2 K \rfloor - 1} \frac{1}{2^{2s} \lfloor K/2^s \rfloor} \sum_{k=0}^{\lfloor K/2^s \rfloor} \left\{ \left[\sum_{j=1}^{2^s} \delta I\left(2^s k + j\right) \right] \left[\sum_{j=1}^{2^s} \delta I\left(2^s (k+1) + j\right) \right] \right\}$$

and

$$C_3 = \sum_{s=0}^{\lfloor \log_2 K \rfloor - 2} \frac{1}{2^{3s} \lfloor K/2^s \rfloor} \sum_{k=0}^{\lfloor K/2^s \rfloor} \left\{ \left[\sum_{j=1}^{2^s} \delta I\left(2^s k + j\right) \right] \left[\sum_{j=1}^{2^s} \delta I\left(2^s (k+1) + j\right) \right] \left[\sum_{j=1}^{2^s} \delta I\left(2^s (k+2) + j\right) \right] \right\}.$$

Here, $\lfloor x \rfloor$ denotes the entire part of number x, and $\delta I(j)$ is the difference between the pixel's intensity $I(j)$ in frame j and its mean intensity averaged over all K recorded frames:

$$\delta I(j) = I(j) - \frac{1}{K}\sum_{k=1}^{K} I(k).$$

The above correlation calculation uses a cascaded averaging of intensities, conflating, on each level s of the cascade, the intensity values of 2^s consecutive frames into one value, thus capturing intensity correlations across all time scales with increasingly coarse-grained temporal resolution. The 2nd and 3rd order SOFI images are then given by all the 2nd and 3rd order correlation values calculated for all pixels. Data analysis was done with a custom-written *Matlab* program, which can be freely obtained from J.E. upon qualified request.

Estimation of image resolution

The spatial resolution of diffraction-limited images (mean intensity z-projections) and sub-diffraction images (2nd and 3rd order SOFI images) were estimated using Fiji [7] and the plugin of the open source algorithm published by Descloux et al. [8].

Determination of microtubule diameters

Microtubule diameters in immunolabeled cells were determined by measuring the full-width-at-half-maximum (FWHM) of the intensity profile perpendicular to straight filaments using home-written analysis tools in Python 3.7. 50 lines equally spaced and perpendicular to a line matching a microtubule were drawn. FWHM values were extracted from a Gaussian fit to the intensity profile and mean FWHM values were calculated for each condition (mean intensity z-projections, SOFI 2nd order and SOFI 3rd order)

Analysis of intensity time traces

Photoinduced fluorophore bleaching was analyzed with open-source program Fiji (1.52a) [7] and home-written routines in Python 3.7. Average image intensities were extracted from movies via ImageJ plugin "Plot Z-axis profile" and saved for further analysis in Python. Intensity traces were normalized to the first frame and the intensities of 40 consecutive frames were averaged. The routine was performed for

>20 cells for all conditions and the average intensity values were plotted with the respective standard deviation.

FRC Analysis

FRC analysis was carried out using the NanoJ plugin in Fiji [9]. To create image pairs for FRC, DNA-PAINT movies were split into overlapping sub-sequences using the Fiji plugin “Slice Keeper” (even and odd frames). To generate the diffraction-limited image, these movies were summed, converted to 8 bit and images were scaled 2-fold without interpolation to match the pixel size of the reconstructed SOFI images. Time series were checked for drift and corrected with NanoJ estimate drift plugin prior to generation of sub-sequences. FRC maps were then created using 10 blocks for good sampling of cell and background areas. The average resolution was then determined by averaging FRC values of the different blocks within the cell borders. Three independent measurements were analyzed per structure. As FRC analysis failed for sum images of TOM20-labeled cells in DNA-PAINT measurements (low signal-to-noise ratio due to HILO illumination), image decorrelation analysis was performed in addition on the images derived from the sub-sequences.

Supplementary Tables

Supplementary Table 1

DNA oligonucleotide docking and imager strands used in this study.

ssDNA oligo	Sequence	Concentration [nM]
P1 docking strand	3´-ATC TAC ATA TT-5´-antibody	
P1 imager strand (8 nt duplex)	5´-AG ATG TAT-3´-AbberiorSTAR635P	20
P4 docking strand	3´-ATC TAA GTA TT-5´-antibody	
P4 imager strand (9nt duplex)	5´-GTA GAT TCA T-3´-Cy3B	5

Supplementary Table 2

Primary antibodies and concentrations used in this study.

Primary antibody	Concentration [µg/ml]
Rabbit-anti-calnexin (#abb22595, Abcam)	5
Mouse-anti-tubulin (#32-2600, Thermo Fisher)	5
Rabbit-anti-TOM20 (#sc-11415, Santa Cruz)	4

Supplementary Figures

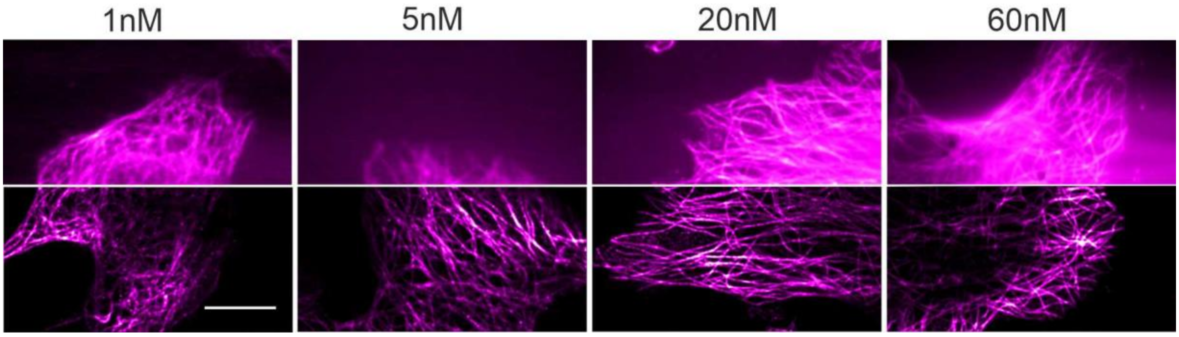

Supplementary Figure 1. SOFI imaging with varying fluorophore-labeled imager strand concentrations. Titration of P1-AbberiorStar635P-labeled imager strands (1 nM to 60 nM) to anti-β-tubulin-labeled U-2 OS cells followed by widefield imaging of side-specific imager fluctuation in HILO mode. Upper panel shows mean intensity projections of 5000 consecutive frames and lower panel display corresponding 2nd order SOFI images (scale bar 10 µm).

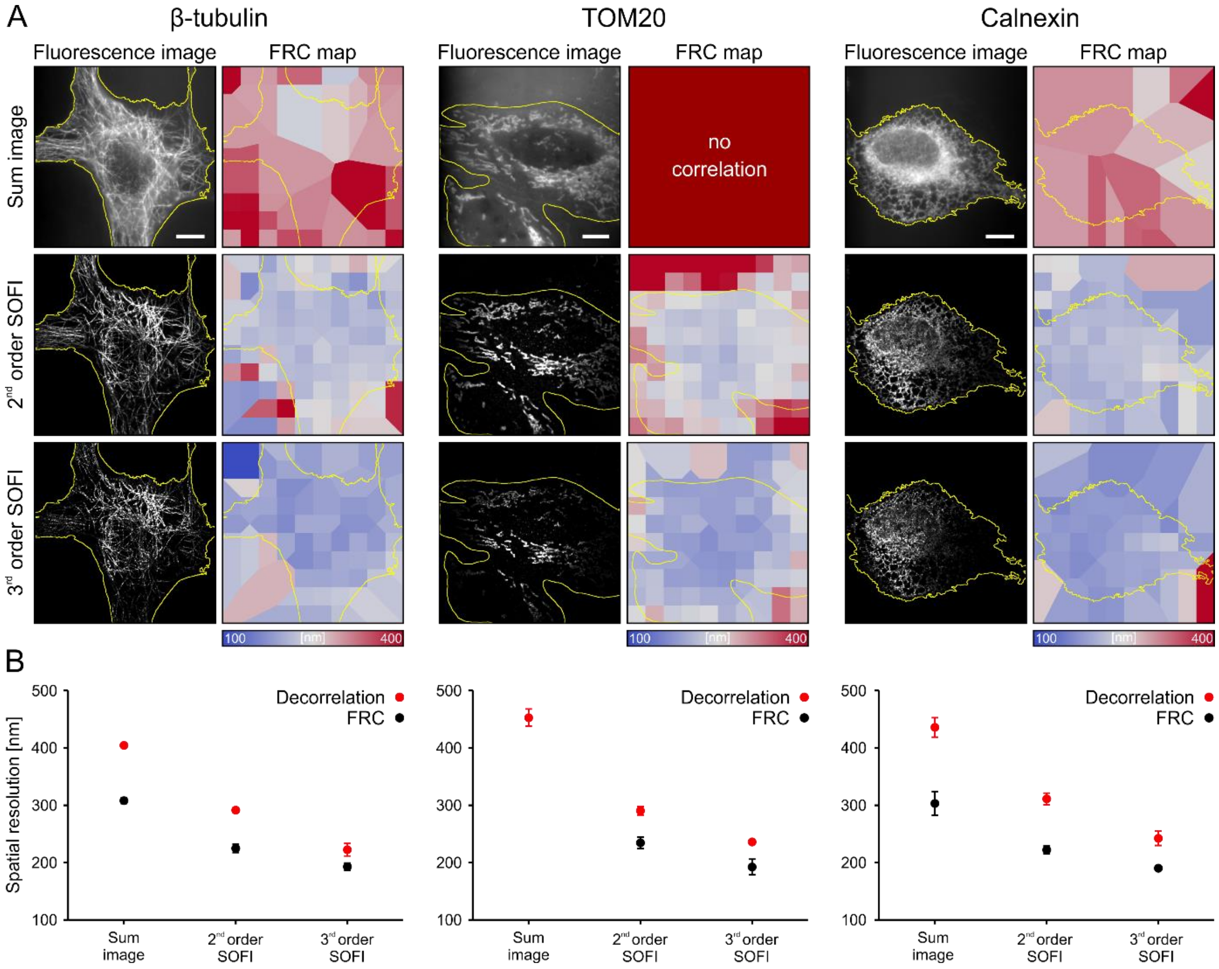

Supplementary Figure 2. Fourier ring correlation (FRC) analysis of SOFI images recorded with exchangeable fluorophore labels. A) Representative cells and corresponding NanoJ FRC maps for all structures imaged. The low signal-to-noise ratio for sum images of TOM20 measurements did not allow for FRC analysis. Yellow lines indicate cell areas that were used to calculate FRC maps (scale bars are 10 µm). B) Quantitative resolution assessment using FRC (black dots) and image decorrelation analysis (red dots). For β-tubulin, we determined FRC values of 308 ± 5 nm (sum image), 225 ± 8 nm (2nd order SOFI) and 193 ± 7 nm (3rd order SOFI) (1.37 and 1.60-fold resolution enhancement). Decorrelation analysis reported a spatial resolution of 404 ± 2 nm, 291 ± 5 nm, and 222 ± 11 nm (1.39 and 1.82-fold resolution enhancement). For TOM20, we determined FRC values of 234 ± 11 nm (2nd order SOFI) and 192 ± 14 nm (3rd order SOFI). Decorrelation analysis reported a spatial resolution of 453 ± 15 nm (sum image), 290 ± 8 nm (2nd order SOFI) and 236 ± 3 nm (3rd order SOFI). For calnexin, we determined FRC values of 303 ± 21 nm (sum image), 222 ± 7 nm (2nd order SOFI) and 190 ± 4 nm (3rd order SOFI). Decorrelation analysis provided respective resolutions of 436 ± 17 nm (sum image), 311 ± 10 nm (2nd order SOFI) and 242 ± 13 nm (3rd order SOFI). Three measurements were analyzed for each cellular structure. Data points in B) represent mean values and error bars the respective standard deviation.

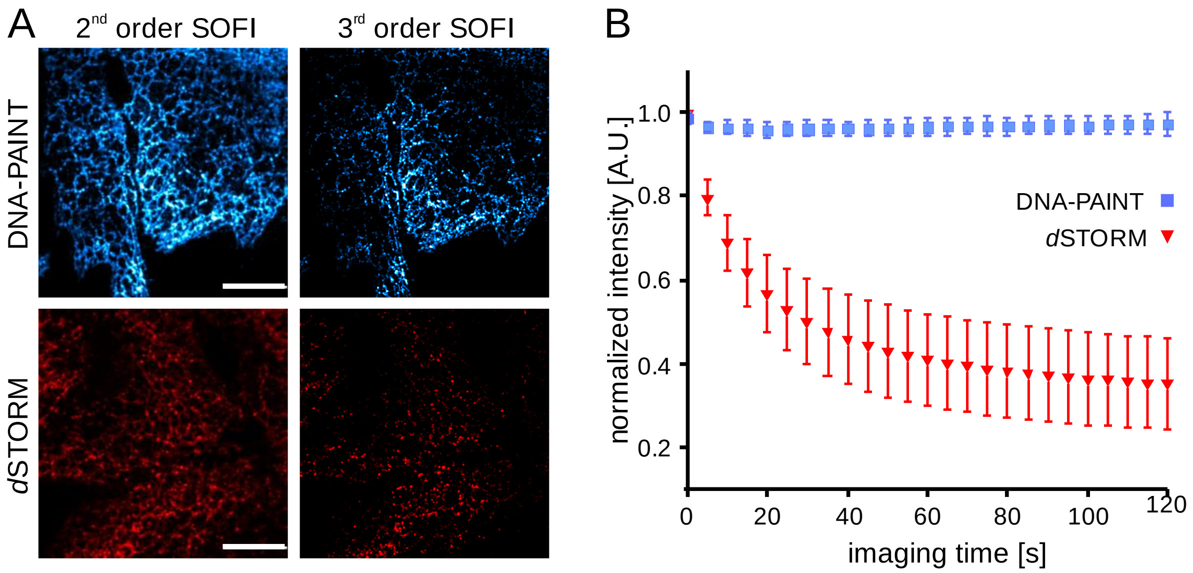

Supplementary Figure 3. Comparison of exchangeable (DNA-PAINT) and permanent (*d*STORM) labels for SOFI. A) Representative 2nd and 3rd order SOFI images of calnexin-labeled U-2 OS cells using DNA-PAINT (upper panel, blue) and *d*STORM (lower panel, red) fluorophore labels to introduce fluorescence fluctuations. Image decorrelation-based analysis of 2nd and 3rd order SOFI images reports an image resolution of 290 ± 8 nm and 233 ± 12 nm for permanent labels and 303 ± 9 nm and 242 ± 9 nm for exchangeable labels. B) Intensity versus time during SOFI experiments using exchangeable (DNA-PAINT, P1-AbberiorStar635P, blue) or permanent (*d*STORM, Alexa Fluor 647, red) labels. Shown are mean intensities ± standard deviation of intensity signals from 20 cells for each condition (scale bars are 10 µm).

References

[1] C. Spahn, F. Hurter, M. Glaesmann, C. Karathanasis, M. Lampe, M. Heilemann, *Angewandte Chemie* **2019**, *58*, 18835-18838.

[2] W. R. Legant, L. Shao, J. B. Grimm, T. A. Brown, D. E. Milkie, B. B. Avants, L. D. Lavis, E. Betzig, *Nature methods* **2016**, *13*, 359-365.

[3] N. S. Deussner-Helfmann, A. Auer, M. T. Strauss, S. Malkusch, M. S. Dietz, H. D. Barth, R. Jungmann, M. Heilemann, *Nano letters* **2018**, *18*, 4626-4630.

[4] K. L. Ellefsen, J. L. Dynes, I. Parker, *PloS one* **2015**, *10*, e0136055.

[5] A. Edelstein, N. Amodaj, K. Hoover, R. Vale, N. Stuurman, *Current protocols in molecular biology* **2010**, *Chapter 14*, Unit14 20.

[6] S. C. Stein, A. Huss, D. Hahnel, I. Gregor, J. Enderlein, *Optics express* **2015**, *23*, 16154-16163.

[7] J. Schindelin, I. Arganda-Carreras, E. Frise, V. Kaynig, M. Longair, T. Pietzsch, S. Preibisch, C. Rueden, S. Saalfeld, B. Schmid, J. Y. Tinevez, D. J. White, V. Hartenstein, K. Eliceiri, P. Tomancak, A. Cardona, *Nature methods* **2012**, *9*, 676-682.

[8] A. Descloux, K. S. Grussmayer, A. Radenovic, *Nature methods* **2019**, *16*, 918-924.

[9] S. Culley, D. Albrecht, C. Jacobs, P.D. Pereira, C. Leterrier, J. Mercer, R. Henriques, *Nature methods* **2018**, *15*, 263-266.
